# Distal radius fractures in the superelderly: an observational study of 8486 cases from the Swedish fracture register

**DOI:** 10.1186/s12877-022-02825-x

**Published:** 2022-02-19

**Authors:** Marcus Sagerfors, Hugo Jakobsson, Ásgerdur Thórdardóttir, Per Wretenberg, Michael Möller

**Affiliations:** 1 Department of Orthopedics and Hand Surgery, Faculty of Medicine and Health, Örebro University, 70182 Örebro, SE Sweden; 2 Institute of Clinical Sciences, Sahlgrenska Academy, University of Gothenburg, Gothenburg, Department of Orthopedics, Sahlgrenska University Hospital, Gothenburg/Mölndal, Sweden

**Keywords:** Distal radius fracture, Patient-related outcome measures, Wrist function

## Abstract

**Background:**

The distal radius fracture (DRF) is the most common fracture in adults. With an ageing population, the number of wrist fractures in the superelderly (≥ 80 years) is expected to rise. Optimal treatment for displaced DRFs remains controversial, especially in the superelderly group. In addition, basic knowledge of the outcome after a DRF in this heterogenic group is lacking. The aim of this study was to study injury characteristics, treatment and outcome of DRFs in superelderly patients using data from a large national register.

**Methods:**

We used prospectively collected data from the Swedish Fracture Register. All distal radius fractures registered between April 2012 and December 2018 in patients ≥ 80 years of age were included. Data on epidemiology, fracture type, trauma mechanism and treatment are registered by the physician treating the patient. Patients are also sent a subjective outcome questionnaire including EQ-5D, EQ-VAS and Short Musculoskeletal Function Assessment questionnaire (SMFA-score) at the time of injury and after 12 months. The 12-month questionnaire was sent to those who had completed the questionnaire at the time of injury. A Mann–Whitney U-test was used to assess differences between treatment methods.

**Results:**

Mean age for this population was 86 years (80–105 years), a majority of the patients were female (86.7%). The dominating injury mechanism was a simple fall (74.6%) in the patient’s residence. The majority of fractures were AO type A (70%) followed by AO type C (20.9%) and type B (8.6%). The incidence of open fractures was significantly higher in females (2.6%) compared to males (1.5%). A majority of the fractures were treated with a cast (87.5%) with volar locking plate as the second most common treatment method (6.6%). Patient-reported outcome measures (PROMs) EQ-5D, EQ-VAS and the Arm Hand Function Index of the SMFA-score deteriorated somewhat one year after injury compared to pre-injury. PROMs did not correlate to treatment with cast or a volar plate.

**Conclusions:**

This nationwide register study provides detailed data on DRFs in the superelderly regarding epidemiology, treatment and self-reported outcome. A good self-reported outcome is possible, but many patients do not recover completely. PROMs did not correlate to type of treatment. The frequency of open fractures was significantly higher in females. The reason for this is unclear but different skin thickness in older males versus females may be one explanation.

## Background

The distal radius fracture (DRF) is the most common fracture in adults, comprising 18% of all fractures in an orthopedic trauma setting [1]. The annual incidence of DRFs in Sweden has been calculated to be 25–28 fractures per 10 000 person-years [2, 3]. Non-displaced or minimally displaced fractures can be managed non-operatively with good results [4], but unstable displaced fractures are generally considered candidates for surgical treatment. In recent years there has been a shift towards more surgery, and the volar locking plate has come to replace previously more common fixation methods such as external fixators and K-wires [3]. There are often considerable differences between younger and older patients regarding trauma mechanism, bone quality, functional demands, and the need for anatomical fracture reduction. Some studies indicate a correlation between functional outcome and the anatomical reduction of the fracture [4, 5], while other studies indicate that this is not the case [6, 7]. For patients over 65 years of age, no correlation between malalignment and patient-reported outcome measures (PROMs) has been shown, and the benefit of reducing DRFs in older, frail persons has limited evidence [8, 9].

In orthopedics, the term “superelderly” has been used to describe persons over 80 years of age [10]. This is the age group with the largest expected relative increase in Sweden, with an expected increase of 50% over a 10-year period [11]. A recent study found that surgical fixation of DRFs in selected superelderly patients can yield a good outcome, but large studies of this growing patient group are generally sparse [12]. The Swedish fracture register (SFR) is a nationwide register prospectively collecting data on orthopedic fractures, patient characteristics, injury, treatment, and PROMs [13]. The purpose of this study was to describe injury characteristics, treatment, and PROMs in superelderly patients, using data from the SFR.

## Methods

This study included all non-pathological DRFs registered in the SFR between January 1^st^ 2012 and December 31^st^ 2018 in patients over 80 years of age. Registration in the SFR is performed prospectively in a web-based program by the attending orthopedic surgeon or junior doctor at the A&E of each affiliated department. Only fractures that have occurred in Sweden and in patients with a Swedish personal identity number are registered. At the beginning of 2015, half of the departments treating fractures in Sweden had joined the SFR, and at the end of 2017, more than 80% of the Swedish population was covered. By 2021, all orthopedic departments in Sweden treating DRFs had joined.

In the SFR, the trauma mechanism is categorized as simple fall, fall from a height, unspecified fall, traffic accident, and other causes. Unspecified falls are those which were not further classified at registration, and can include a simple fall or fall from a height. The trauma mechanism is further specified as high-energy or low-energy. It is up to the registering doctor to categorize the trauma energy level, as there is no strict guideline to distinguish high-energy from low-energy trauma. Fracture type is classified according to the Muller AO/OTA system [14], which has been shown to have a generally high level of accuracy and validity as well as a moderate accuracy of classification of DRFs in the SFR [15–18]. An AO/OTA classification manual with schematic images of the different fracture groups and accompanying written explanations is used in the online classification process. Injury location is categorized as the patient’s residence (including institutional housing), on a street/road, in a public place, or at an unspecified place.

Treatment is divided into non-operative and operative, the latter of which is further divided into volar plate, Kirchner wires (k-wires), external fixator, dorsal plate, and other methods including combination treatments such as external fixator plus K-wires, external fixator plus a volar plate, spanning plate, and so on. The level of experience of the operating surgeon is categorized as resident in orthopedic surgery, resident assisted by an orthopedic surgeon, orthopedic trauma surgeon (with > 50% trauma in daily practice), other orthopedic subspecialty, or hand surgeon.

PROMs used in the SFR include validated Swedish translations of the EQ-5D, the EQ-VAS, and two indexes (arm/hand function and bother) from the Short Musculoskeletal Function Assessment (SMFA) [19, 20]. Questionnaires are sent to the patient a short time after the fracture. The patient is asked to report their health status in the week before the fracture occurred (recall technique). One reminder is sent out after 4 weeks. The 1-year follow-up questionnaires are sent only to those patients who complete the initial questionnaires.

The EQ-5D is one of the most common generic questionnaires used to measure health-related quality of life (HRQoL) for a range of conditions and treatments [21]. Respondents report their health in five dimensions (mobility, self-care, usual activities, pain/discomfort, and anxiety/depression), with one question used to measure each dimension. Prior to February 2019, the SFR used the EQ-5D 3L, which has three levels of severity, but from then onwards it has used the five-level version (EQ-5D 5L). Thus, some fractures from 2018 were first assessed with EQ-5D 3L and then followed up with EQ-5D 5L. The EQ-5D also contains a visual analog scale (EQ VAS) component for an overall assessment of the respondent’s health. The EQ-5D health state can be summarized into a single index (EQ-5D Index) by applying a formula attaching specific weights to each severity level in each dimension. These indices are commonly anchored at 1 (full health) and 0 (health state as bad as being dead).

The SMFA is designed to measure the functional status of patients with a broad range of musculoskeletal injuries and disorders. The Swedish version has been demonstrated to be reliable and sensitive to changes over time [20]. It comprises two parts: a dysfunction index with 34 items, and a bother index with 12 items. The dysfunction items are grouped into four categories: daily activities, emotional status, function of the arm and hand, and mobility. A transformation formula gives a final score ranging from 0 to 100, with higher scores indicating poorer function. Mortality is included in the SFR, and is updated daily by a link to the Swedish Tax Agency. One-year mortality was calculated.

### Statistics

Nominal variables are presented as proportions of the registered fractures, and scale variables are presented as mean and standard deviation (SD). Change over time was assessed with the Wilcoxon sign rank test due to a non-normal distribution (Shapiro–Wilk test, data not shown). Due to the descriptive nature of the study, extensive additional statistical testing was not undertaken. The Mann–Whitney U-test was used to assess differences between different treatment methods. A chi-squared test was used to assess differences between men and women regarding frequency of open fractures. A *P*-value < 0.05 was considered statistically significant.

### Results

A total of 50 035 DRFs were registered between April 1^st^ 2012 and December 31^st^ 2018. Of these fractures, 8486 occurred in patients aged 80 and older. The mean age in this group was 86 years (range 80–105). A majority of the cases were female (86.7%), and the left side was slightly more commonly fractured (56.7% of the cases). A simple fall was the most common cause of injury (74.6%), while a fall from height represented 10.7% of the injuries.

**Fig. 1:**
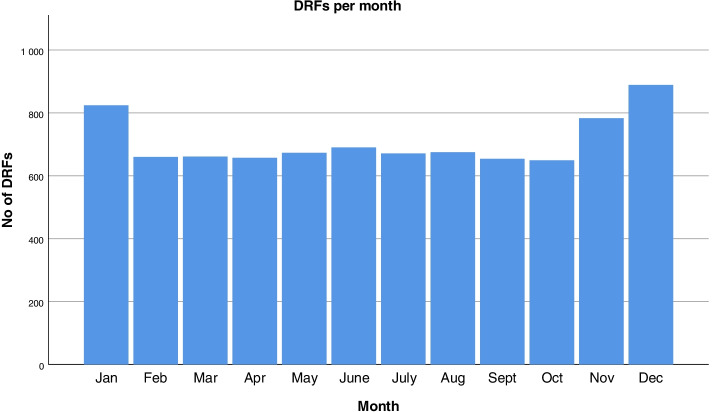
Number of distal radius fractures per month

A majority of the fractures were AO type A (70%), with type C fractures being the second most common fracture type (20.9%) followed by type B (8.6%). Open fractures constituted 2.5% of the DRFs. Among men, 1.5% of the fractures were open, while the corresponding figure among women was 2.6% (*p* = 0.025). Women with open fractures had a mean age of 86.7 years, while men with open fractures had a mean age of 84.3 years. Of the open fractures that were classified according to the Gustilo-Anderson classification, a majority (1.1%) were type I, followed by types II (0.5%) and III (0.1%) [22]. Most fractures took place in the patient’s residence or accommodation (57.8%, *n* = 4904/8486), while 7.9% occurred on a street/road (*n* = 673/8486), 3.6% (*n* = 303/8486) in a public place, and 27.6% (*n* = 2344/8486) in other unspecified places (Table 2). November, December, and January were the months with the highest frequency of DRFs (Fig. 1).

The primary treatment was non-surgical for 87.5% (*n* = 7423/8486) of cases and surgical for 12.5% (*n* = 1063/8486). The most common surgical intervention was a volar plate (6.6%, *n* = 558/8486) followed by K-wires (1.1%, *n* = 93/8486) and external fixator (0.4%, 33/8486). Volar plates were used in 4.8% of the AO type A cases, 8.2% of type B cases, and 11.8% of type C cases (Table 1).

**Table 1:** Type of treatment *n (*%)

Treatment	All types	AO type A	AO type B	AO type C
Cast	7423 (87.5)	5361 (90.2)	636 (86.9)	1393 (78.7)
Volar plate	558 (6.6)	286 (4.8)	60 (8.2)	209 (11.8)
K-wires	93 (1.1)	69 (1.2)	5 (0.7)	19 (1.1)
External fixator	33 (0.4)	15 (0.3)	3 (0.4)	15 (0.8)
Dorsal plate	7 (0.1)	3 (0.1)	3 (0.4)	1 (0.1)
Other	408 (4.8)			

Other treatments include combinations of treatment such as external fixator + K-wires, external fixator + volar plate, bridge plate, or volar plate + K-wires

For 558 of the cases treated with a volar plate, the experience level of the operating surgeon was specified; among these, most fractures were operated by orthopedic surgeons or orthopedic trauma surgeons (Table 2).

**Table 2:** Experience level of surgeon performing treatment with volar plate

Operating surgeon	*n*
Resident	98
Resident assisted by orthopedic surgeon	29
Orthopedic surgeon	239
Orthopedic trauma surgeon	155
Hand surgeon	11
Other	9
Missing	17

A majority of the patients reported no problems with mobility, self-care, usual activities, and anxiety/depression before the fracture, whereas problems with pain/discomfort were more common. Moderate problems with mobility, usual activities, and pain/discomfort were relatively more common one year after the fracture (Table 3).

**Table 3:** Distribution of reported problems with respect to all EQ-5D dimensions before and one year after the fracture *n* (%)

Factor	**EQ-5D pre-fracture**			**EQ-5D 1 year after the fracture**		
	**No problems**	**Moderate problems**	**Severe problems**	**No problems**	**Moderate problems**	**Severe problems**
Mobility	2093 (55.4)	1671 (44.2)	17 (0.4)	1082 (51.7)	982 (46.9)	30 (1.4)
Self-care	2743 (72.3)	836 (22.0)	217 (5.7)	1624 (76.4)	383 (18.0)	120 (5.6)
Usual activity	2423 (64.6)	877 (23.4)	450 ()12.0	1372 (65.3)	509 (24.2)	219 (10.4)
Pain/discomfort	1430 (37.8)	2135 (56.4)	222 (5.9)	778 (36.9)	1199 (56.8)	134 (6.3)
Anxiety/depression	2384 (62.9)	1278 (33.7)	126 (3.3)	1343 (63.6)	695 (32.9)	72 (3.4)

All PROMS (EQ-5D Index, EQ-VAS, and arm/hand function and bother indices of the SMFA) deteriorated significantly one year after the fracture compared to before the fracture (Table 4).

**Table 4:** Patient-reported outcome measures, mean (standard deviation)

	**Pre-injury**	**1 year after injury**	***p*-value**
EQ-5D Index	0.69 (0.31)	0.68 (0.31)	< 0.001
EQ-5D VAS	72.1 (22.0)	66.9 (23.3)	< 0.001
SMFA arm/hand function index	20.1 (24.1)	23.4 (24.0)	< 0.001
SMFA bother index	21.1 (20.3)	24.8 (22.1)	< 0.001

EQ-5D Index ranges from 0 to 1, where 1.0 is optimal outcome. EQ-5D VAS ranges from 0 100, where 100 is optimal outcome/health status. The SMFA indexes range from 0 to 100, where higher scores indicate poorer outcome. Statistical significance was assessed using the Wilcoxon sign rank test

The number of respondents to the EQ-5D was 3671/8486 at the time of injury and 2019/8486 at the 1-year follow-up. No significant difference was seen between patients treated with a cast and patients treated with a volar plate for these outcome measures. The 1-year mortality was 11.8%.

## Discussion

Based on our literature search, this study represents the largest cohort study of DRFs in the superelderly. The majority of DRFs occurred in women due to a low-energy fall in the patient’s residence. The female/male ratio of 87/13 was substantially higher than that reported in a British study, but also higher than reported in other studies [2, 23, 24]. This is likely due to the relatively higher proportion of postmenopausal women in the superelderly group compared to other studies. The incidence of open fractures was 2.5%, which is substantially higher than the previously-reported incidence of 0.7% open fractures in a superelderly cohort [25]. This may indicate that open DRFs are more common than other open fractures in the superelderly population. We found a significantly higher incidence of open DRFs in superelderly women compared to men, and this was also substantially higher than in another register study which reported a 1.2% incidence of open fractures in an adult population [24]. Our findings are however in line with the finding of Court-Brown that superelderly women had a higher incidence of open fractures compared to men [25]. The reason for this is unclear, but the mean age of the women was somewhat higher than that of the men, and it is possible that the women were more frail. On the other hand, men have a shorter life expectancy, and sustain low-energy fractures at a younger age than women [26]. Another explanation could be that male skin is thicker than female skin, as has been demonstrated for all anatomical locations [27], and the male dermis is estimated to be 1.8 times thicker than the female dermis [28]. In addition, the ageing process alters the mechanical properties of the skin as well as the elastin and collagen organization [29].

The most common place of injury was the patient´s residence/accommodation (57.8%), which differs substantially from the 33% found in another study on DRF epidemiology in Sweden [24]. The reason for this is likely the sedentary lifestyle of the superelderly compared to younger people. The relative incidence of type A and C fractures was higher and the relative incidence of type B fractures lower compared to the findings in another study [24]. One explanation for this difference could be that the superelderly group likely has an inferior bone quality compared to the general population, resulting in lower incidence of shearing type B partial articular fractures and instead a higher relative incidence of type A and C fractures [30].

The 1-year mortality in the present study was 11.8%, which is substantially higher than previously reported after a DRF, but likely due to the high mean age of this selected population [24]. In comparison, the 1-year mortality after a proximal femur fracture was reported to be 26% in a cohort of patients with a mean age of 83 years [31].

November, December, and January were the months with the highest frequency of DRFs. This is in line with a previous study which found that the DRF incidence was twice as high in January compared to May, most likely due to less favorable conditions including ice and slippery streets during the winter in Sweden [24]. November, December, and January aside, the DRF incidence was very equally distributed over the year.

Fractures were classified according to the AO/OTA classification system, which has a proven high accuracy and validity [16]. We found that the majority (70%) of the DRFs were extra-articular (AO type A), while 8.6% were partially articular (type B) and 20.9% were completely intra-articular. For the distal radius, some studies indicate that the AO/OTA classification seems to be the most reliable classification system, but with only a fair inter-observer reliability for the type of fracture, suggesting that the subgroup classification (A1, A2, etc.) is of limited value [32, 33]. Given the challenges with subgroup classifications, we focused on fracture type rather than type of subgroup. The classification of DRFs in the SFR has a moderate accuracy as demonstrated in previous studies [15, 33]. The fractures in this study were classified by the treating surgeon according to AO/OTA subgroup, thus reflecting the classification made in real-life conditions by attending orthopedic surgeons, some with limited experience.

In our study, 12.5% of the DRFs were treated surgically. This is a lower proportion than that observed in a Swedish national register study of DRF epidemiology, where the proportion of surgically treated fractures in 2010 was 20% [3]. A majority of the fractures regardless of fracture type were managed non-surgically. Of the DRFs in superelderly patients treated surgically, the vast majority were treated with a volar plate. This finding is in line with previous studies reporting that plate fixation is the preferred surgical method in several countries [3, 34, 35]. Volar plates were used in a considerably higher proportion of the type C fractures compared to the type A fractures (11.8% and 4.8% respectively). One explanation could be that unacceptable joint incongruence as well as fracture instability remaining after closed reduction may result in a decision to operate.

Patient satisfaction is sometimes described as a combination of subjective and social-cultural feelings in addition to behavioral, cognitive, and psychological influences [36], and so it is paramount to understand what the metric truly captures in patients following a DRF. Moderate problems with mobility, usual activities, and pain/discomfort were relatively more common one year after the fracture compared to before the fracture. The clinical relevance of this finding is unclear. One confounder in using a generic PROM like the EQ-5D may be that the group of superelderly are likely to experience some limitations regarding mobility and self-care due to their advanced age [37]. PROMs were assessed using the EQ-5D, the EQ-VAS, and the arm/hand function and bother indexes of the SMFA, and the reported outcome deteriorated significantly one year after the fracture compared to before the fracture. However, there was no significant difference between the PROMs when comparing treatment with a cast to a volar plate. As treatment was not randomized, we cannot conclude which treatment is optimal for a DRF in the superelderly. This observation is in accordance with the findings of Egol et al. comparing closed reduction and casting with operative treatment in patients over 65 years. Operative intervention yielded superior radiographic outcome but similar functional status at the two-year follow-up [38].

The EQ-5D has been shown to display an acceptable to good responsiveness in patients with a DRF [19]. However, the fact that the EQ-5D and SMFA are not PROMs designed specifically for upper extremity disorders could limit their ability to capture deterioration in wrist function, compared to more disease-specific PROMs like the Disabilities of the Arm, Shoulder and Hand (DASH) and Patient-Rated Wrist Evaluation (PRWE) instruments. On the other hand, both the EQ-5D and the SMFA reflect the general well-being of the patient, which is arguably an important outcome measure. A study by Saving et al. comparing volar plate versus non-operative treatment for a dorsally dislocated DRF in patients > 70 years of age found significantly better grip strength, PRWE score, and DASH score at the one-year follow-up for patients treated with a volar plate, but no significant difference in EQ-5D [39]. In addition, the reliability of PROMs may differ between young and old study participants, but data on this are largely lacking.

A retrospective study of 76 superelderly patients treated with a volar plate found that surgical fixation can yield a good functional outcome [40]. Other studies have found no significant relationship between malalignment after a DRF and functional outcome in older patients [8, 41]. Yet another study found no association between either fracture subtype or the level of anatomical restoration and the 1-year outcome after surgical management of a DRF in adults treated operatively [42].

Treatment was not given based on common guidelines but rather based on local traditions and personal preferences. The first national guidelines on treatment for DRFs in Sweden were only launched very recently [43]. A previous study demonstrated a large variation in operative management of DRFs between Swedish healthcare regions [44]. At the end of 2017, the SFR was expected to cover more than 80% of the Swedish population, which gives a good representation of DRF treatment in Sweden [24].

The large number of cases represents a major strength of our study and provides prospectively registered data on a national level, reducing the risk of bias due to varying local treatment traditions and epidemiological and sociodemographic differences. Data are registered in the SFR in a pre-specified systematic way regarding patient, fracture, and treatment characteristics, providing detailed data on orthopedic fractures and fracture treatment. To our knowledge, this study is unique in size and detail regarding DRFs in the superelderly. The presence of a high-energy or low-energy trauma was not assessed according to a specific score such as the Injury Severity Score, which is a limitation [45]. On the other hand, the fact that high-energy trauma was not clearly defined means that it represents what the average general orthopedic surgeon treating DRFs believes is a high-energy trauma. The lack of full national coverage is a limitation, although coverage improved substantially during the study period.

The response rate for the PROMs represents an obvious limitation of all register studies of this type, but to some degree is compensated by the large number of fractures included in the study. In addition, another study from the SFR found that the non-responders in the SFR reported similar function compared to the initial responders regarding PROMs [46]. Given a 1-year mortality of almost 12% for this cohort, we believe the response rate in our study is acceptable. Completeness of fracture data is important and has been investigated in SFR recently concluding that the SFR constitutes a complete, accurate and efficient source of information [47]. As for fractures that are not admitted under orthopedic teams, the organization of Swedish healthcare is such that all fractures are treated at accident and emergency departments and followed up at the orthopedic departments, all of which had joined the SFR by 2021. Another limitation in this study is the risk of recall bias, as patients at inclusion were asked to fill out the questionnaires for their pre-injury status as of the week before the injury. The radiographic 1-year outcome is not classified in the SFR, which is another limitation of this study. However, the relation between radiographic outcome and patient-reported outcome is still unclear, especially for older patients [8, 41].

## Conclusions

The findings from this study indicate that choice of treatment (volar plate or non-operative treatment) has limited correlation with PROMs after a DRF in the superelderly population. Overall, PROMs deteriorated significantly 1 year after a DRF compared to before the fracture. Open fractures were significantly more frequent in superelderly women than in superelderly men, and also more frequent than reported in previous studies. Further studies, preferably multicenter randomized controlled trials (RCT) comparing different treatment modalities and with relevant outcome measures, are indicated to improve treatment for this growing group of patients. If a fracture register is available, as is the case in Sweden, these studies can preferably be conducted as register-based RCTs.

## Data Availability

The datasets used and/or analyzed during the current study are available from the corresponding author on reasonable request.

## Abbreviations

AO/OTA: Arbeitsgemeinschaft für Osteosynthesefragen/Orthopedic Trauma Association
DASH: Disabilities of the Arm, Shoulder and Hand
DRF: Distal radius fracture
EQ-5D: EuroQoL 5 dimensions;
EQ VAS: EuroQoL Visual analog scale
K-wires: Kirchner wires
PROM: Patient reported outcome measure
PRWE: Patient-rated wrist evaluation
SMFA-score: Short Musculoskeletal Function Assessment
SFR: Swedish Fracture Register

## References

[1] Court-Brown CM, Caesar B (2006). Epidemiology of adult fractures: A review. Injury.

[2] Brogren E, Petranek M, Atroshi I (2007). Incidence and characteristics of distal radius fractures in a southern Swedish region. BMC Musculoskelet Disord.

[3] Mellstrand-Navarro C, Pettersson HJ, Tornqvist H, Ponzer S (2014). The operative treatment of fractures of the distal radius is increasing: results from a nationwide Swedish study. The bone & joint journal.

[4] McQueen M, Caspers J (1988). Colles fracture: does the anatomical result affect the final function?. J Bone Joint Surg Br.

[5] Karnezis IA, Panagiotopoulos E, Tyllianakis M, Megas P, Lambiris E (2005). Correlation between radiological parameters and patient-rated wrist dysfunction following fractures of the distal radius. Injury.

[6] Goldfarb CA, Rudzki JR, Catalano LW, Hughes M, Borrelli J (2006). Fifteen-year outcome of displaced intra-articular fractures of the distal radius. J Hand Surg Am.

[7] Anzarut A, Johnson JA, Rowe BH, Lambert RG, Blitz S, Majumdar SR (2004). Radiologic and patient-reported functional outcomes in an elderly cohort with conservatively treated distal radius fractures. J Hand Surg Am.

[8] Grewal R, MacDermid JC (2007). The risk of adverse outcomes in extra-articular distal radius fractures is increased with malalignment in patients of all ages but mitigated in older patients. J Hand Surg Am.

[9] Beumer A, McQueen MM (2003). Fractures of the distal radius in low-demand elderly patients: closed reduction of no value in 53 of 60 wrists. Acta Orthop Scand.

[10] Court-Brown CM, Clement N (2009). Four score years and ten: an analysis of the epidemiology of fractures in the very elderly. Injury.

[11] Statistics Sweden, SE-104 51 Stockholm S. Demographic reports 2018:1 The future population of Sweden 2018–2070. 2018. https://www.scb.se/publication/33003.

[12] Saving J, Severin Wahlgren S, Olsson K, Enocson A, Ponzer S, Sköldenberg O (2019). Nonoperative Treatment Compared with Volar Locking Plate Fixation for Dorsally Displaced Distal Radial Fractures in the Elderly: A Randomized Controlled Trial. J Bone Joint Surg Am.

[13] Bergh C, Möller M, Ekelund J, Brisby H. 30-day and 1-year mortality after skeletal fractures: a register study of 295,713 fractures at different locations. Acta Orthop. 2021;92(6):739–45. 10.1080/17453674.2021.1959003. Epub 2021 Jul 26. 10.1080/17453674.2021.1959003 PMC8635666 34309486

[14] Marsh JL, Slongo TF, Agel J, Broderick JS, Creevey W, DeCoster TA (2007). Fracture and dislocation classification compendium - 2007: Orthopaedic Trauma Association classification, database and outcomes committee. J Orthop Trauma.

[15] Bergvall M, Bergdahl C, Ekholm C, Wennergren D (2021). Validity of classification of distal radial fractures in the Swedish fracture register. BMC Musculoskelet Disord.

[16] Juto H, Möller M, Wennergren D, Edin K, Apelqvist I, Morberg P (2016). Substantial accuracy of fracture classification in the Swedish Fracture Register: Evaluation of AO/OTA-classification in 152 ankle fractures. Injury.

[17] Wennergren D, Ekholm C, Sundfeldt M, Karlsson J, Bhandari M, Möller M (2016). High reliability in classification of tibia fractures in the Swedish Fracture Register. Injury.

[18] Wennergren D, Stjernström S, Möller M, Sundfeldt M, Ekholm C (2017). Validity of humerus fracture classification in the Swedish fracture register. BMC Musculoskelet Disord.

[19] Rundgren J, Enocson A, Mellstrand Navarro C, Bergström G (2018). Responsiveness of EQ-5D in Patients With a Distal Radius Fracture. Hand (New York, NY).

[20] Ponzer S, Skoog A, Bergström G (2003). The Short Musculoskeletal Function Assessment Questionnaire (SMFA): cross-cultural adaptation, validity, reliability and responsiveness of the Swedish SMFA (SMFA-Swe). Acta Orthop Scand.

[21] Devlin NJ, Brooks R (2017). EQ-5D and the EuroQol Group: Past, Present and Future. Appl Health Econ Health Policy.

[22] Gustilo RB, Anderson JT (1976). Prevention of infection in the treatment of one thousand and twenty-five open fractures of long bones: retrospective and prospective analyses. J Bone Joint Surg Am.

[23] Stirling ERB, Johnson NA, Dias JJ (2018). Epidemiology of distal radius fractures in a geographically defined adult population. J Hand Surg Eur.

[24] Rundgren J, Bojan A, Mellstrand Navarro C, Enocson A (2020). Epidemiology, classification, treatment and mortality of distal radius fractures in adults: an observational study of 23,394 fractures from the national Swedish fracture register. BMC Musculoskelet Disord.

[25] Court-Brown CM, Biant LC, Clement ND, Bugler KE, Duckworth AD, McQueen MM (2015). Open fractures in the elderly. The importance of skin ageing Injury.

[26] Court-Brown CM, Aitken SA, Ralston SH, McQueen MM (2011). The relationship of fall-related fractures to social deprivation. Osteoporos Int.

[27] Millington GWM, Graham-Brown RAC. Skin and Skin Disease Throughout Life. Rook's Textbook of Dermatology. 2010;1:1-29. 10.1002/9781444317633

[28] Makrantonaki E, Brink TC, Zampeli V, Elewa RM, Mlody B, Hossini AM (2012). Identification of biomarkers of human skin ageing in both genders. Wnt signalling - a label of skin ageing? PLoS One.

[29] Makrantonaki E, Bekou V, Zouboulis CC (2012). Genetics and skin aging. Dermatoendocrinol.

[30] Falaschi P, Marques A, Giordano S. Osteoporosis and fragility in elderly patients. In: Orthogeriatrics: The Management of Older Patients with Fragility Fractures. Cham: Springer; 2021. p. 35-32. 10.1007/978-3-030-48126-1.

[31] Wolf O, Mukka S, Ekelund J, Möller M, Hailer NP (2021). How deadly is a fracture distal to the hip in the elderly? An observational cohort study of 11,799 femoral fractures in the Swedish Fracture Register. Acta Orthop.

[32] Waever D, Madsen ML, Rolfing JHD, Borris LC, Henriksen M, Nagel LL (2018). Distal radius fractures are difficult to classify. Injury.

[33] Plant CE, Hickson C, Hedley H, Parsons NR, Costa ML (2015). Is it time to revisit the AO classification of fractures of the distal radius? Inter- and intra-observer reliability of the AO classification.. The bone & joint journal..

[34] Mattila VM, Huttunen TT, Sillanpaa P, Niemi S, Pihlajamaki H, Kannus P. Significant change in the surgical treatment of distal radius fractures: a nationwide study between 1998 and 2008 in Finland. J Trauma. 2011;71(4):939–42; discussion 42–3. 10.1097/TA.0b013e3182231af9 21986738

[35] Azad A, Kang HP, Alluri RK, Vakhshori V, Kay HF, Ghiassi A (2019). Epidemiological and Treatment Trends of Distal Radius Fractures across Multiple Age Groups. Journal of wrist surgery.

[36] Brokelman RB, Haverkamp D, van Loon C, Hol A, van Kampen A, Veth R (2012). The validation of the visual analogue scale for patient satisfaction after total hip arthroplasty. Eur Orthop Traumatol.

[37] Collard RM, Boter H, Schoevers RA, Oude Voshaar RC (2012). Prevalence of frailty in community-dwelling older persons: a systematic review. J Am Geriatr Soc.

[38] Egol KA, Walsh M, Romo-Cardoso S, Dorsky S, Paksima N (2010). Distal radial fractures in the elderly: operative compared with nonoperative treatment. J Bone Joint Surg Am.

[39] Saving J, Enocson A, Ponzer S, Mellstrand Navarro C. External Fixation Versus Volar Locking Plate for Unstable Dorsally Displaced Distal Radius Fractures-A 3-year Follow-Up of a Randomized Controlled Study. J Hand Surg Am. 2018. 10.1016/j.jhsa.2018.09.015 30420192

[40] Heng BQH, Kang YC, Lim JXY, Chee KG (2020). Epidemiology of Distal Radius Fixations and Functional Outcomes in the Superelderly Population. The journal of hand surgery Asian-Pacific volume.

[41] Clement ND, Duckworth AD, Court-Brown CM, McQueen MM. Distal radial fractures in the superelderly: does malunion affect functional outcome? ISRN Orthop. 2014;2014:189803. 10.1155/2014/189803 PMC4045364 24967123

[42] Sagerfors M, Lundqvist E, Bjorling P (2020). Combined Plating of Intra-Articular Distal Radius Fractures, a Consecutive Series of 74 Cases. Journal of wrist surgery.

[43] https://nationelltklinisktkunskapsstod.se/vardprogramochvardforlopp Accessed Oct 1st 2021

[44] Saving J, Ponzer S, Enocson A, Mellstrand Navarro C (2018). Distal radius fractures-Regional variation in treatment regimens. PLoS One..

[45] Husum H, Strada G (2002). Injury Severity Score versus New Injury Severity Score for penetrating injuries. Prehosp Disaster Med.

[46] Juto H, Gärtner Nilsson M, Möller M, Wennergren D, Morberg P (2017). Evaluating non-responders of a survey in the Swedish fracture register: no indication of different functional result. BMC Musculoskelet Disord.

[47] Bergdahl C, Nilsson F, Wennergren D, Ekholm C, Möller M (2021). Completeness in the Swedish Fracture Register and the Swedish National Patient Register: An Assessment of Humeral Fracture Registrations. Clin Epidemiol.

